# LINC00511 as a prognostic biomarker for human cancers: a systematic review and meta-analysis

**DOI:** 10.1186/s12885-020-07188-3

**Published:** 2020-07-22

**Authors:** Yannick Luther Agbana, Manzama-Esso Abi, Yueli Ni, Guohang Xiong, Jing Chen, Fang Yun, Zihan Yi, Qiao Zhang, Zhe Yang, Yingmin Kuang, Yuechun Zhu

**Affiliations:** 1 Department of Biochemistry and Molecular Biology, Kunming Medical University, Kunming, 650500 Yunnan Province China; 2 Department of Microbiology and Immunology, Kunming Medical University, Kunming, 650500 Yunnan Province China; 3 Department of Pathology, The First Affiliated Hospital of Kunming Medical University, Wuhua District, Kunming, 650032 Yunnan Province China; 4 Department of Organ Transplantation, The First Affiliated Hospital of Kunming Medical University, Wuhua District, Kunming, 650032 Yunnan Province China

**Keywords:** LINC00511, Prognostic biomarker, Survival, Meta-analysis, TCGA, Cancer

## Abstract

**Background:**

Long intergenic non-coding RNA 00511 (LINC00511) is highly expressed in diverse cancers and has a correlation with poor clinical outcomes for cancer patients. In view of contradictory data among published data, we aim to evaluate the prognostic role of LINC00511 for cancer patients.

**Methods:**

In the present study, a meta-analysis of related studies has been performed to investigate the prognostic significance of LINC00511 in cancer patients. Relevant studies published before December 22, 2019 were systematically searched online in PubMed, EMBASE, Web of Science, and the Cochrane Library databases. The relationship between LINC00511 expression and cancer patients’ survival, including overall survival (OS), disease-free survival (DFS)/relapse-free survival (RFS) and progression-free survival (PFS), was evaluated using pooled hazard ratios (HRs) with their corresponding 95% confidence intervals (CIs). The association between LINC00511 expression and clinicopathological features was assessed using odd ratios (ORs) and their corresponding 95% CIs.

**Results:**

A total of 14 eligible studies with 1883 patients were enrolled in the present meta-analysis. The results demonstrated that elevated expression of LINC00511 was significantly associated with poor OS (HR = 2.62; 95% CI: 2.00–3.45; *p* <  0.001), PFS (HR = 1.80; 95% CI: 1.29–2.51; *p* = 0.001) and DFS/RFS (HR = 2.90; 95% CI: 1.04–8.12; *p* = 0.04). Additionally, High LINC00511 expression was associated with large tumor size (OR = 3.10; 95% CI: 1.97–4.86; *p* <  0.00001), lymph node metastasis (OR = 3.11; 95% CI: 2.30–4.21; *p* <  0.00001), advanced clinical stage (OR = 3.95; 95% CI: 2.68–5.81; *p* <  0.00001), distant metastasis (OR = 2.39; 95% CI: 1.16–4.93; *p* = 0.02), and disease recurrence (OR = 4.62; 95% CI: 2.47–8.65; *p* <  0.00001). Meanwhile, no correlation was found between LINC00511 expression and age, gender, and histological grade. These findings were consolidated by the results of bioinformatics analysis.

**Conclusions:**

Based on our findings, LINC00511 may serve as a novel prognostic biomarker for cancer patients.

## Background

Long non-coding RNAs (lncRNAs) are referred to as the non-protein coding portion of the transcriptome with length of more than 200 nucleotides. They represent, together with small noncoding RNAs (< 200 nucleotides), the biggest part of the transcriptome, since only 1 to 3% of the transcriptome can code for protein synthesis [1–3]. Long noncoding RNAs are involved in several biological processes including cell proliferation, cell differentiation, cell cycle progression, cell apoptosis and metastasis [4–10]. Notably, they were identified as oncogenes or tumor suppressors, prognostic and diagnostic biomarkers and as therapeutic targets [11–13]. For instance, Tang and colleagues reviewed the implication of lncRNAs in colorectal cancer progression and figured out their potential clinical applications as novel diagnostic and prognostic biomarkers and therapeutic targets [14].

Long intergenic non-coding RNA 00511 (LINC00511) is a 2265-bp lncRNA mapped to chromosome 17q24.3 with five exons. Recent studies found that LINC00511 is overexpressed in various type of cancers including breast cancer, ovarian cancer, liver cancer, pancreatic cancer, lung cancer and glioma. LINC00511 is an oncogene which plays a negative regulatory role in cell proliferation, cell cycle progression, apoptosis, invasion, migration, metastasis and chemoresistance [15–23]. Noteworthily, overexpression of LINC00511 was shown to be a predictor for cancer prognosis [16, 18, 20, 22, 24–28]. However, the results of the studies were not consistent. For instance, the studies of Deng et al. [27], Zhao et al. [20] and Wang et al. [18] showed no correlation between the expression level of LINC00511 and tumor size; in contrast the studies of Sun et al. [22], Yu et al. [25] and Zhang et al. [26] demonstrated an association between LINC00511 expression and tumor size. Similarly, Lu et al. [16] detected significant correlation between LINC00511 overexpression and lymph node metastasis in breast cancer, whereas the study conducted by Zhang et al. [26] provided a contradictory result. In view of these contradictory outcomes, we conducted a systematic review and meta-analysis to evaluate the prognostic role of LINC00511 for cancer patients. Next, we validated our results by using The Cancer Genome Atlas (TCGA) and Genotype-Tissue Expression (GTEx) datasets.

## Methods

### Literature search strategies

The present study was performed according to the Preferred Reporting Items for Systematic Reviews and Meta-Analysis (PRISMA) [29]. Relevant studies published before December 22, 2019 were systematically searched online in PubMed, EMBASE, Web of Science and the Cochrane Library databases. The following search terms were used in different combinations: (LINC00511 OR “long intergenic noncoding RNA 00511” OR “long intergenic non-protein coding RNA 511”) AND (cancer OR tumor OR tumour OR carcinoma OR neoplasm OR adenoma OR sarcoma OR malignancy). The reference lists of the full-text articles were also checked to find relevant studies that pointed out the relation between LINC00511 expression and clinical outcomes. No language restriction was applied.

### Inclusion and exclusion criteria

The eligibility criteria were as follows: (1) the study subjects were patients with any type of cancer; (2) the studies investigated association between LINC00511 expression levels and clinical outcomes in cancer; (3) patients were separated into LINC00511 high expression level and LINC00511 low expression level groups; (4) the LINC00511 expression levels were measured by quantitative method (e.g., real-time reverse transcription polymerase chain reaction (qRT-PCR)); and (5) sufficient information and data were provided to calculate a hazard ratios (HRs) or odd ratios (ORs) and their corresponding 95% confidence intervals (CIs). Exclusion criteria were as follows: (1) duplicate publications; (2) reviews and meta-analyses; (3) studies without patient samples; (4) studies not relevant to cancer, LINC00511 or prognosis.

### Publication quality assessment

The quality of included studies was assessed using the Newcastle-Ottawa Scale (NOS). Three main categories have been considered including selection, comparability and outcome, and the stars rating system has been used. The scores of NOS were ranged from 0 star (lowest score) to 9 stars (highest score). A study with a NOS score higher than 5 was considered as a high-quality study [30, 31].

### Data extraction

Two investigators reviewed all eligible studies independently and extracted the following data: the first author’s name, year of publication, country of origin, the tumor type, sample size, cut-off value, detection method, HRs and corresponding 95% CIs, and clinicopathological features. Any controversial issue was resolved by discussion. In case HR value was not provided, Engauge Digitizer version 11.2 (http://markummitchell.github.io/engauge-digitizer/) was used to extract data from Kaplan-Meier curve. The extracted data were then used to estimate HRs and 95% CIs by using the *HR calculations spreadsheet* provided by Tierney et al. [32].

### Bioinformatics analysis

We used GEPIA, a web server for cancer and normal gene expression profiling and interactive analyses, to perform bioinformatics analysis. GEPIA uses the data from both TCGA (The Cancer Genome Atlas) and GTEx (Genotype-Tissue Expression) to perform several analyses. The datasets were computed by the UCSC Xena project based on a standard pipeline [33, 34]. Therefore, we directly use GEPIA to perform our analyses. GEPIA used Kaplan-Meier method and log-rank test for the survival analysis and one-way ANOVA for the gene expression analysis. First, the expression levels of LINC00511 in the different cancer types involved in our meta-analysis was analyzed by matching TCGA and GTEx data. Next, overall survival and disease-free survival curves were retrieved.

### Statistical analysis

All statistical analyses were performed with RevMan 5.3 software (Cochrane community, https://community.cochrane.org/help/tools-and-software/revman-5/revman-5-download/) and STATA 15.0. The pooled HRs and their 95% CIs were used to evaluate the association of LINC00511 expression with the cancer patients’ survival. Odd ratios (ORs) analyses were performed to assess the correlation between LINC00511 expression and clinicopathological parameters. The heterogeneity between studies results was examined by Cochran’s Q test and Higgins I-squared (I^2^) statistic. In the absence of heterogeneity (*p* ≥ 0.10 and/or I^2^ < 50%), the fixed-effect model was used; otherwise the random-effect model was applied [35, 36]. Potential publication bias was evaluated using the funnel plot, Egger’s test and Begg’s test. Sensitivity analysis was also performed to assess the impact of individual study on the pooled effect [37]. A *p*-value less than 0.05 was considered to be statistically significant.

## Results

### Characteristics of included studies

**Fig. 1:**
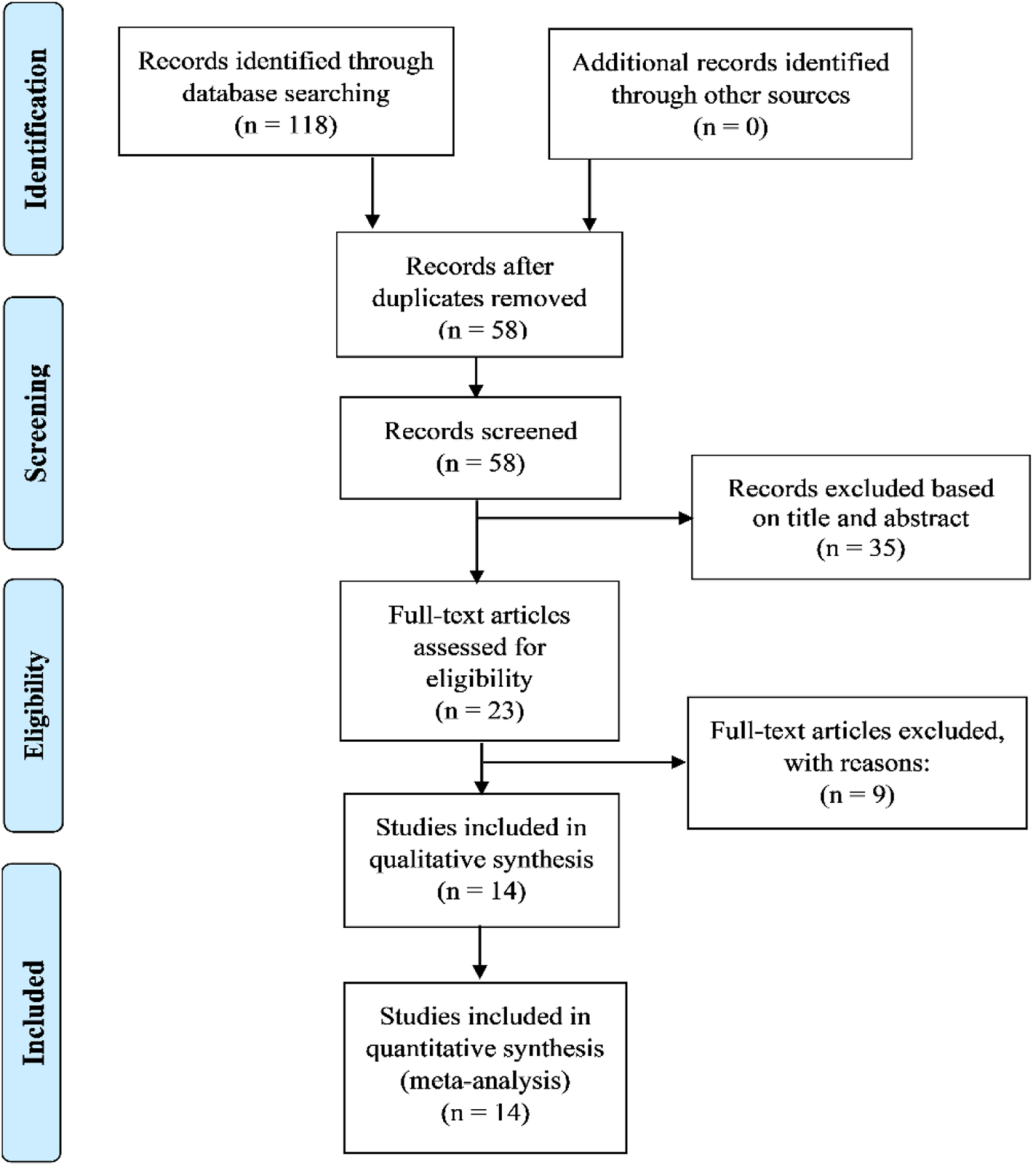
Flowchart of the literature search strategy

A flowchart of the literature search strategy is shown in Fig. 1. According to the described search strategy, 118 articles were retrieved. Sixty articles were excluded based on duplication criteria. The 58 articles remained, underwent an initial screening. On the basis of title and abstract, 35 articles (non-cancer publications, non-LINC00511 publications, reviews and meta-analyses, retracted article, articles without prognosis and clinical data) were excluded. A total of 23 full-text articles were eventually assessed for eligibility. After reading full-text articles, 9 articles were excluded for reasons: articles without prognosis and clinical data in respect to LINC00511, not available full-text article and non-patient samples. At the end, 14 articles were used for the quantitative synthesis [16–22, 24–28, 38, 39]. The 14 studies involved a total of 1883 patients and all came from China. The sample size of included studies ranged from 36 to 412 patients and the type of cancer included non-small-cell lung cancer, pancreatic cancer, breast cancer, cervical cancer, hepatocellular carcinoma, ovarian cancer, and brain tumors. The articles included in the present study were all written in English and published between 2016 and 2019. The expression level of LINC00511 was mainly determined by qRT-PCR. The cut-off values included mean, median and the P25 value. In our study, the quality of all included studies is high (NOS score > 5). The overall characteristics of included studies are summarized in Table 1.

**Table 1:** Characteristics of the studies included in the meta-analysis

First author	Year	Country	Tumor type	Sample size (n)	Gender (Male/Female) (n)	LINC00511 expression (High/low) (n)	Detection method	Tumor stage	Cut-off value	Survival analysis	Outcome	Quality score	Data extraction method
Sun C.-C.	2016	China	NSCLC	124	58/66	93/31	qRT-PCR	I-IV	P25 value	Univariate/Multivariate	OS/CP	8	Direct
Zhao X.	2017	China	PDAC	140	78/62	102/38	qRT-PCR	I-III	Not available	Univariate/Multivariate	OS/PFS/CP	7	Direct
Lu G.	2018	China	BC	39	0/39	23/16	qRT-PCR	I-IV	Mean	Kaplan-Meier	OS/CP	7	Indirect
Mao B.-D.	2019	China	CC	84	0/84	49/35	qRT-PCR	I1a-II2b	Median (0.996)	Kaplan-Meier	OS/RFS/CP	7	Indirect
Wang R.-P.	2019	China	HCC	127	76/51	64/63	qRT-PCR	I-IV	Median	Univariate/Multivariate	OS/CP	8	Direct
Zhang J.	2019	China	BC	70	0/70	24/46	qRT-PCR	I-IV	Mean	Kaplan-Meier	OS/CP	8	Indirect
Liu L.	2019	China	BC	98	0/98	49/49	qRT-PCR	I-IV	Median	Kaplan-Meier	OS/CP	7	Indirect
Yu C.-L.	2019	China	CC	92	0/92	46/46	qRT-PCR	I-IV	Not available	Univariate/Multivariate	OS/CP	8	Direct
Deng H.	2019	China	ccRCC	49	21/28	25/24	qRT-PCR	I-IV	Median	Kaplan-Meier	OS/CP	6	Indirect
Zhu F.-Y.	2019	China	NSCLC	57	28/29	29/28	qRT-PCR	I-IV	Median	Kaplan-Meier	OS/CP	8	Direct
Du X.	2019	China	GBM	36	24/12	18/18	qRT-PCR	Not available	Median	Kaplan-Meier	OS/PFS/CP	7	Indirect
Wang J.	2019	China	OC	378	Not available	189/189	qRT-PCR	Not available	Median	Univariate	OS	7	Direct
Wang Z.	2019	China	PC	177	Not available	134/43	RNA sequencing expression data	Not available	Not available	Kaplan-Meier	OS	7	Direct
Li C.	2019	China	GL	412	Not available	206/206	qRT-PCR	Not available	Not available	Kaplan-Meier	OS	6	Indirect

*Abbreviations*: *NSCLC* Non-small-cell lung cancer, *PDAC* Pancreatic ductal adenocarcinoma, *BC* Breast cancer, *CC* Cervical cancer, *HCC* hepatocellular carcinoma, *ccRCC* Clear cell renal cell carcinoma, *GBM* Glioblastoma multiforme, *OC* Ovarian cancer, *PC* Pancreatic cancer, *GL* Glioma, *qRT-PCR* Quantitative real time polymerase chain, *OS* Overall survival, *PFS* Progression-free survival, *RFS* Relapse-free survival, *DFS* Disease-free survival, *CP* Clinicopathological parameters

### Association between LINC00511 expression and cancer patients’ survival

**Fig. 2:**
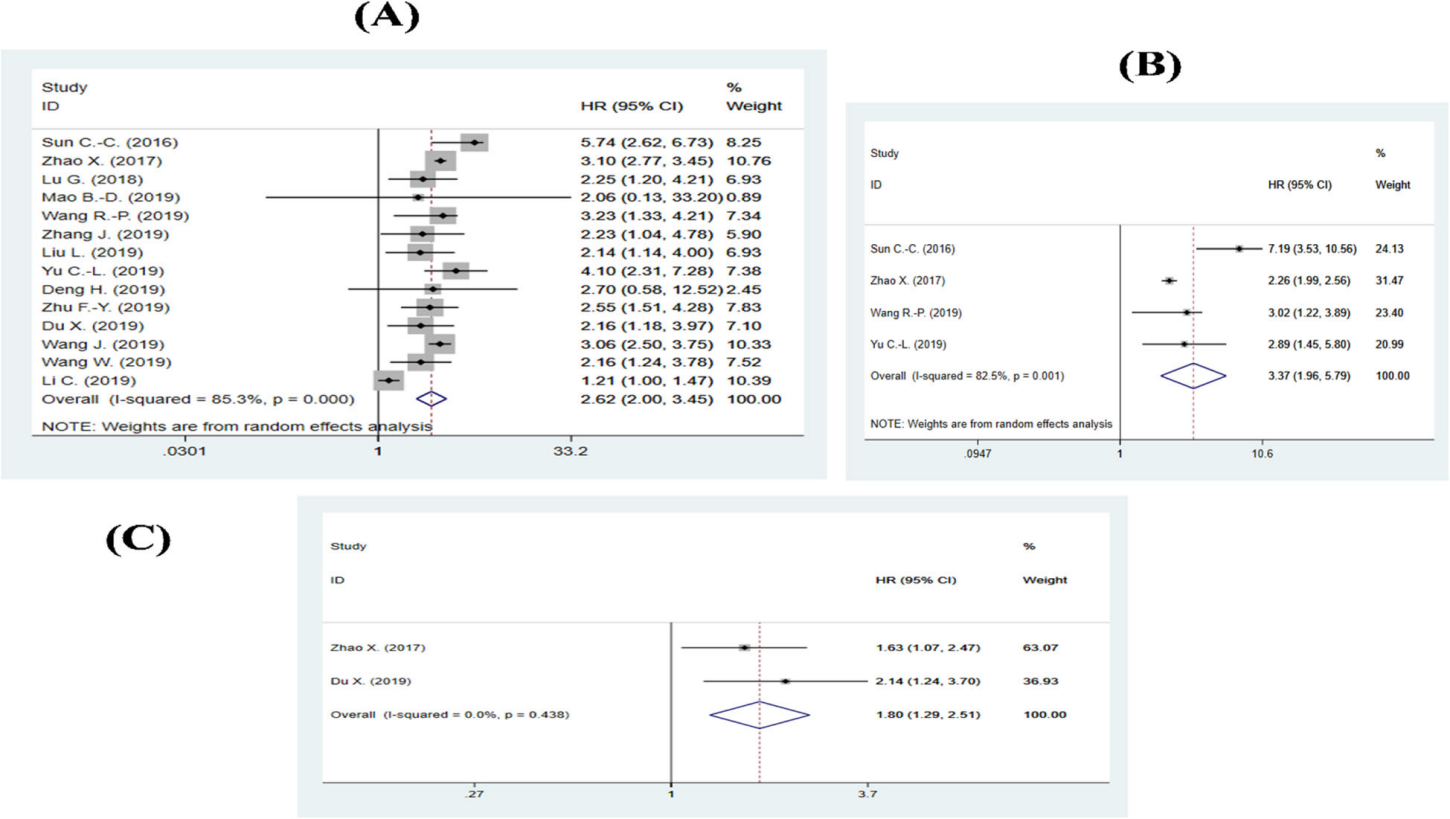
Forest plots of combined analyses on the association of survival with LINC00511 expression. **a** Forest plot of OS analysis, **b** Forest plot of meta-analysis of the independent predictive value of LINC00511 for OS, and **c** forest plot of PFS analysis

Elevated LINC00511 expression was significantly predictive of poor overall survival (HR = 2.62; 95% CI: 2.00–3.45; *p* <  0.001). A high heterogeneity was observed among the studies (P_H_ < 0.001; I^2^ = 85.3%), so the random-effect model was used (Fig. 2a). In addition, high LINC00511 expression has been proven to have an independent prognostic value in four studies by processing the multivariate analysis [18, 20, 22, 25]. As previously reported [40], we conducted the meta-analysis of these studies and found that high LINC00511 expression may serve as an independent predictor for poor overall survival prognosis in cancers (HR = 3.37; 95% CI: 1.96–5.79; *p* < 0.001; I^2^ = 82.5%; P_H_ = 0.001; Fig. 2b).

The relationship between LINC00511 expression and cancer progression was investigated by combining progression-free survival (PFS) studies. The results, from only two studies with available related data, demonstrated that high LINC00511 expression was predicted to be associated significantly with worse PFS (HR = 1.80; 95% CI: 1.29–2.51; *p* = 0.001; I^2^ = 0%; P_H_ = 0.44; Fig. 2c). In addition, one study found that high LINC00511 may be associated with worse relapse-free survival (HR = 2.90; 95% CI: 1.04–8.12; *p* = 0.04).

**Fig. 3:**
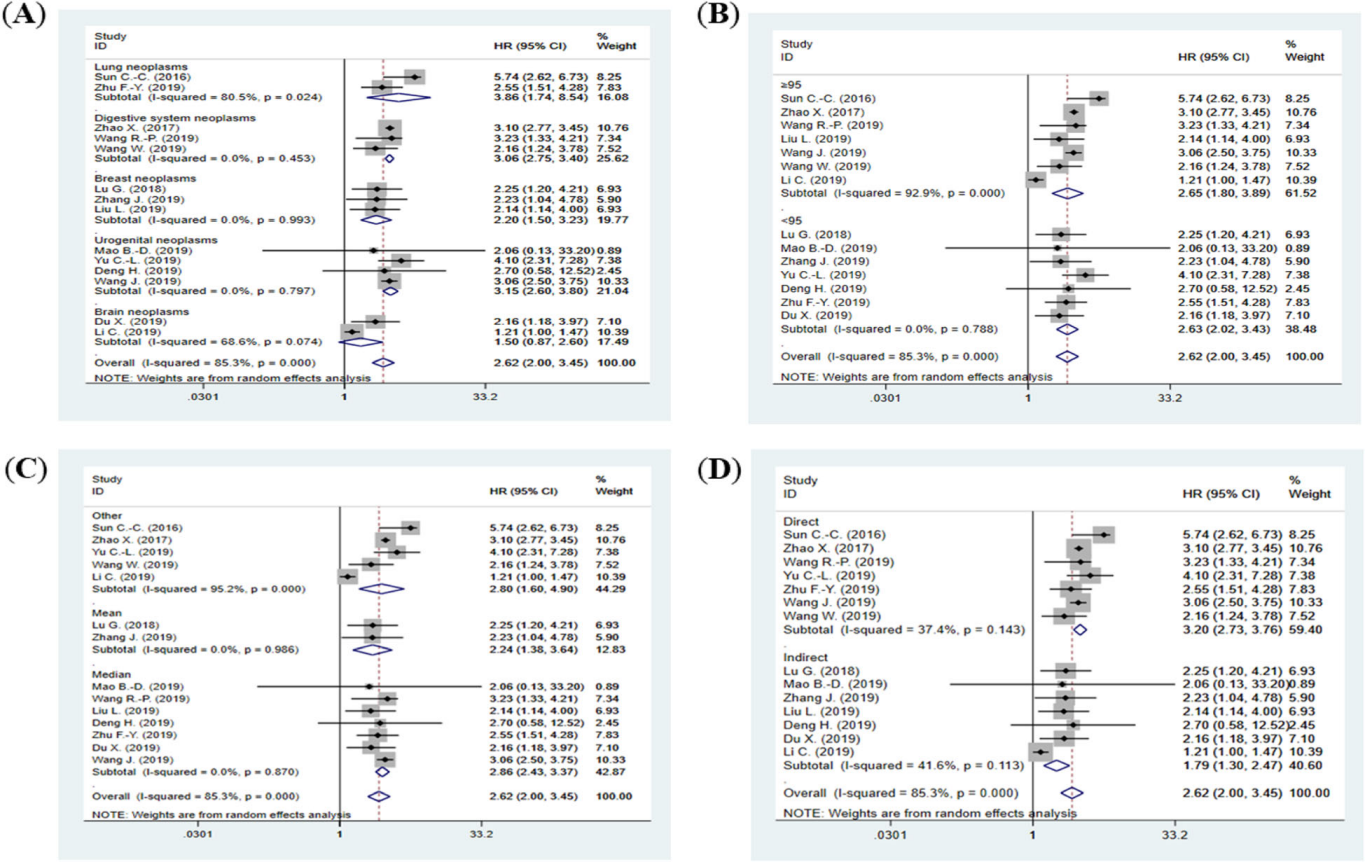
Forest plots of the association between LINC00511 expression and overall survival in subgroups based on **a** cancer types; **b** sample size; **c** different cut-off value and **d** different extraction method

Subgroup meta-analyses based on cancer types (according to NCBI’s medical subject headings (MeSH) [41]) (Fig. 3a), sample size (Fig. 3b), cut-off value (Fig. 3c) and data extraction method (Fig. 3d) were conducted. The results showed that high LINC00511 expression was significantly associated with poor overall survival in lung neoplasms (HR = 3.86; 95% CI: 1.74–8.54; *p* = 0.001; I^2^ = 80.5%; P_H_ = 0.02), digestive system neoplasms (HR = 3.06; 95% CI: 2.76–3.40; *p* < 0.001; I^2^ = 0%; P_H_ = 0.45), breast neoplasms (HR = 2.20; 95% CI: 1.50–3.23; *p* < 0.001; I^2^ = 0%; P_H_ = 0.99) and urothelial neoplasms (HR = 3.15; 95% CI: 2.60–3.80; *p* < 0.001; I^2^ = 0%; P_H_ = 0.80). Regarding the brain neoplasms subgroup, despite the relatively high HR, the relationship cannot be considered robust because the *p*-value is higher than 0.05 (HR = 1.50; 95% CI: 0.87–2.60; *p* = 0.15; I^2^ = 68.6%; P_H_ = 0.07). In respect to sample size subgroups, there was significant correlation between high LINC00511 expression and poor overall survival prognosis of cancer patients in both large sample size (*n* ≥ 95) (HR = 2.65; 95% CI: 1.81–3.89; *p* < 0.001; I^2^ = 92.9%; P_H_ < 0.001) and small sample size (*n* < 95) (HR = 2.63; 95% CI: 2.02–3.43; *p* < 0.001; I^2^ = 0%, P_H_ = 0.79). Concerning the cut-off value subgroups analysis, whether the median was used as cut-off value (HR = 2.86; 95% CI: 2.43–3.38; *p* < 0.001; I^2^ = 0%; P_H_ = 0.87) or the mean as cut-off value (HR = 2.24; 95% CI: 1.38–3.64; *p* = 0.001; I^2^ = 0%, P_H_ = 0.99) or other cut-off values were used (HR = 2.80; 95% CI: 1.60–4.90; *p* < 0.001; I^2^ = 95.2%; P_H_ < 0.001), high LINC00511 expression correlated with poor overall survival in cancer patients. Similarly, the subgroup meta-analysis based on the data extraction method showed that whether the data were extracted directly from the literature (HR = 3.21; 95% CI: 2.73–3.76; *p* < 0.001; I^2^ = 37.4%; P_H_ = 0.14) or indirectly (HR = 1.80; 95% CI: 1.30–2.47; *p* < 0.001; I^2^ = 41.6%; P_H_ = 0.11), high LINC00511 expression was significantly associated with poor overall survival in cancer patients. Of note, the heterogeneity is likely to come from diverse sources.

### Association between LINC00511 expression and clinicopathological features

The meta-analysis of the correlation between LINC00511 expression and clinicopathological parameters are summarized in Table 2. The pooled odds ratio (OR) values revealed significant association between high LINC00511 expression and large tumor size (OR = 3.10; 95% CI: 1.97–4.86; *p* < 0.00001; I^2^ = 50%; P_H_ = 0.03), lymph node metastasis (OR = 3.11; 95% CI: 2.30–4.21; *p* < 0.00001; I^2^ = 42%; P_H_ = 0.08), advanced clinical stage (OR = 3.95; 95% CI: 2.68–5.81; *p* < 0.00001; I^2^ = 0%; P_H_ = 0.48), distant metastasis (OR = 2.39; 95% CI: 1.16–4.93; *p* = 0.02; I^2^ = 21%; P_H_ = 0.28), and disease recurrence (OR = 4.62; 95% CI: 2.47–8.65; *p* < 0.00001; I^2^ = 0%; P_H_ = 0.66). Meanwhile, the results showed no statistically significant correlation between LINC00511 expression and age, gender, and histological grade.

**Table 2:** Meta-analyses of the correlation between LINC00511 expression and clinicopathological features

Variables	Number of studies	Number of patients	Model	OR		Heterogeneity	
				95% CI	*p*-value	I^2^ (%)	P_H_
Gender (Male vs. Female)	6	498	Random	0.98 (0.53–1.85)	0.96	58	0.04
Age							
≥45 vs. < 45	1	84	–	0.66 (0.28–1.59)	0.36	–	–
≥50 vs. < 50	6	462	Fixed	0.69 (0.47–1.01)	0.06	0	0.98
≥60 vs. < 60	1	140	–	0.72 (0.34–1.52)	0.39	–	–
≥65 vs. < 65	2	173	Fixed	0.59 (0.29–1.20)	0.15	0	0.89
Histological grade (poor vs. well and moderate)	8	725	Fixed	1.34 (0.97–1.85)	0.07	0	0.75
Histological tumor type (adenocarcinoma vs. squamous cell carcinoma)	3	300	Random	2.80 (0.58–13.59)	0.20	71	0.03
Tumor size (large vs. small)	11	916	Random	3.10 (1.97–4.86)	**< 0.00001**	50	0.03
Lymph node metastasis (positive vs. negative)	10	880	Fixed	3.11 (2.30–4.21)	**< 0.00001**	42	0.08
Clinical stage (late vs. early)	8	643	Fixed	3.95 (2.68–5.81)	**< 0.00001**	0	0.48
Distant metastasis (Yes vs. No)	3	215	Fixed	2.39 (1.16–4.93)	**0.02**	21	0.28
Recurrence (Yes vs. No)	3	274	Fixed	4.62 (2.47–8.65)	**< 0.00001**	0	0.66

*Abbreviations*: *OR* Odd ratio, *P*_*H*_*P*-value of heterogeneity

### Publication bias

**Fig. 4:**
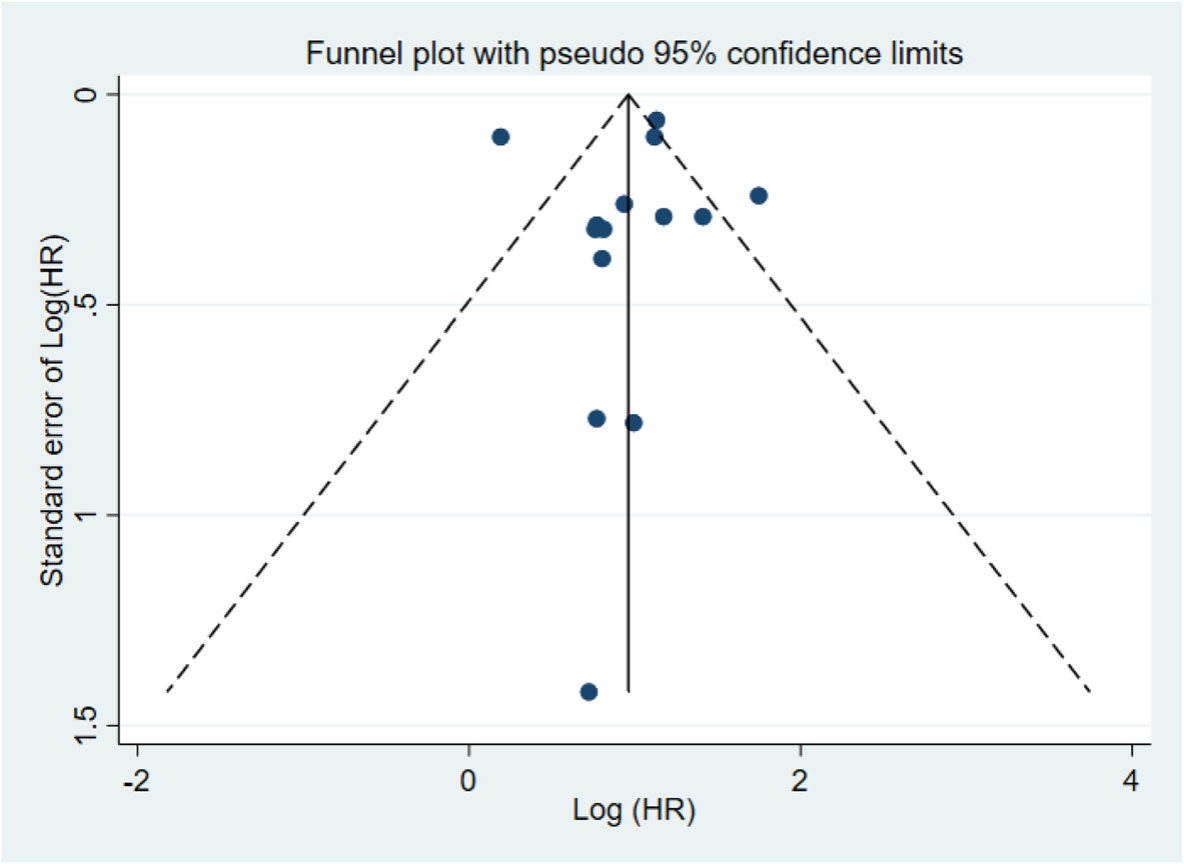
Funnel plot of publication bias based on Overall survival (OS)

Funnel plot analysis, Egger’s test and Begg’s test were performed to evaluate potential publication bias. There was no obvious asymmetry on the funnel plot of OS (Fig. 4) and the tests of publication bias (Begg’s test: *p* = 0.161; Egger’s test: *p* = 0.630) indicated that no publication bias existed in studies reported OS.

### Sensitivity analysis

**Fig. 5:**
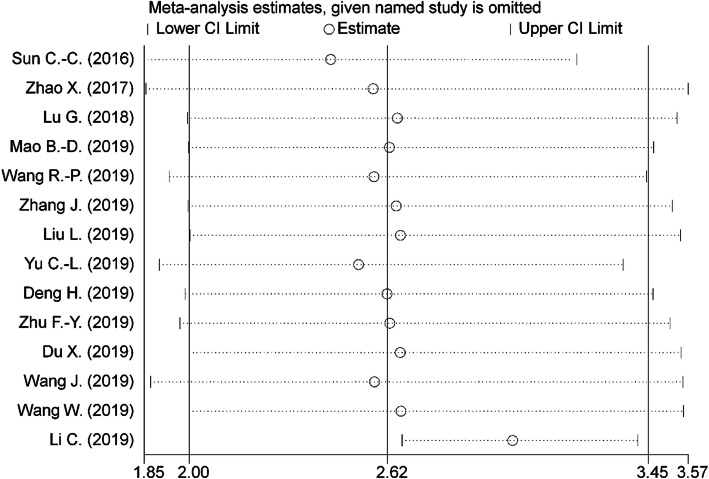
Sensitivity analysis of pooled HR for overall survival

The sensitivity analysis was performed by omitting each individual study to test the stability of the pooled result of the association between LINC00511 expression and OS. As shown on Fig. 5, when “Li C. (2019)” was omitted, the pooled result oscillated. Subsequently, we evaluated the pooled HR after removing “Li C. (2019)” and found that elevated LINC00511 expression still predicted worse OS (HR = 3.06; 95% CI: 2.80–3.33; *p* < 0.001; I^2^ = 14.8%; P_H_ = 0.30). Therefore, the influential study didn’t alter the significance of the pooled result, sustaining the reliability of our pooled result.

### Datasets analyses by GEPIA

**Fig. 6:**
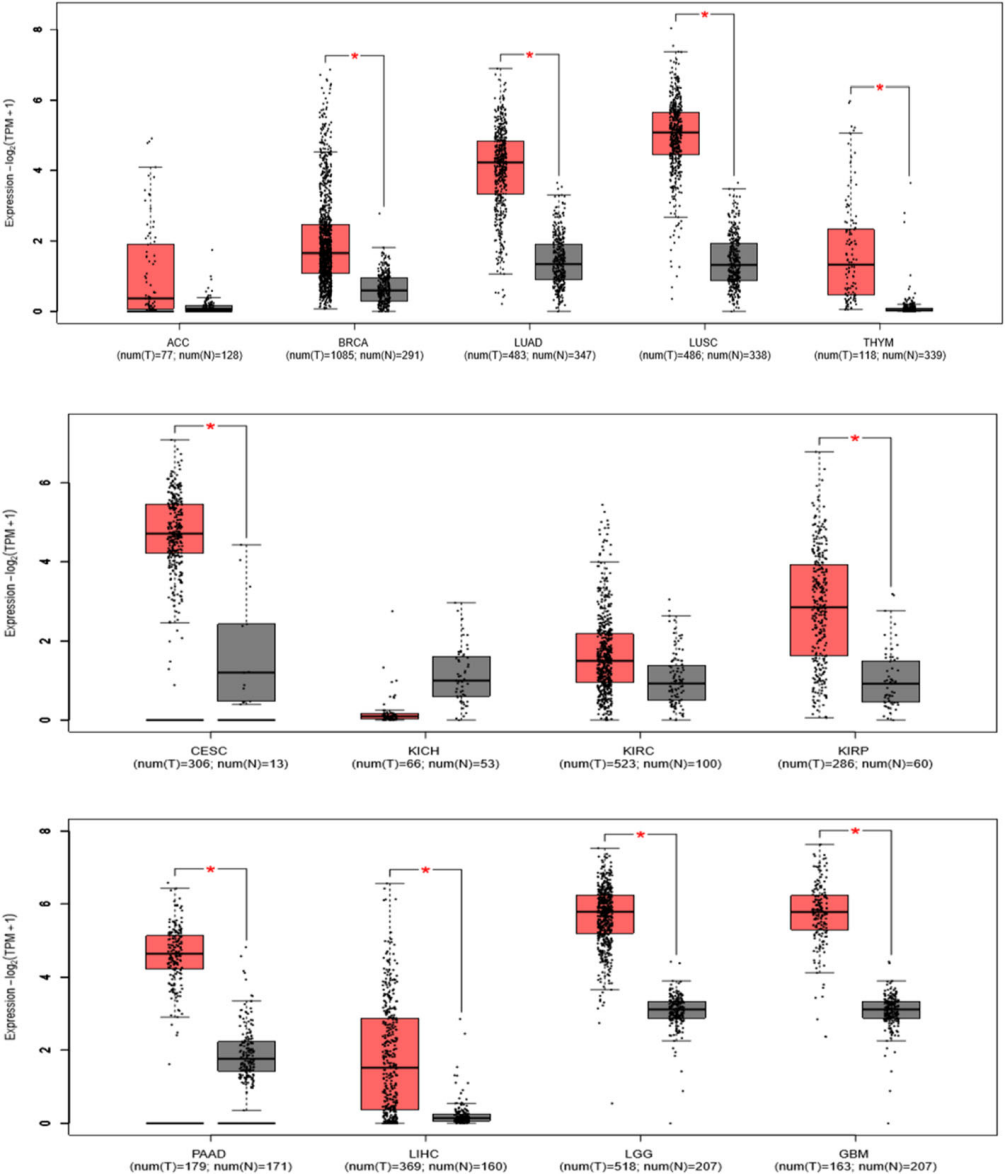
Validation of the expression levels of LINC00511 in TCGA normal and GTEx datasets: “*” indicates log_2_FC value > 1 and *p*-value < 0.01. TCGA, The Cancer Genome Atlas; GTEx, Genotype-Tissue Expression; ACC, adrenocortical carcinoma; THYM, thymoma; LGG, brain lower grade glioma; GBM, glioblastoma multiforme; LUAD, lung adenocarcinoma; LUSC, lung squamous cell carcinoma; PAAD, pancreatic adenocarcinoma; BRCA, breast invasive carcinoma; CESC, cervical squamous cell carcinoma; LIHC, liver hepatocellular carcinoma; KIRC, Kidney renal clear cell carcinoma; KIRP, Kidney renal papillary cell carcinoma; KICH, Kidney Chromophobe; TPM, transcripts per million; T, tumor; N, normal

**Fig. 7:**
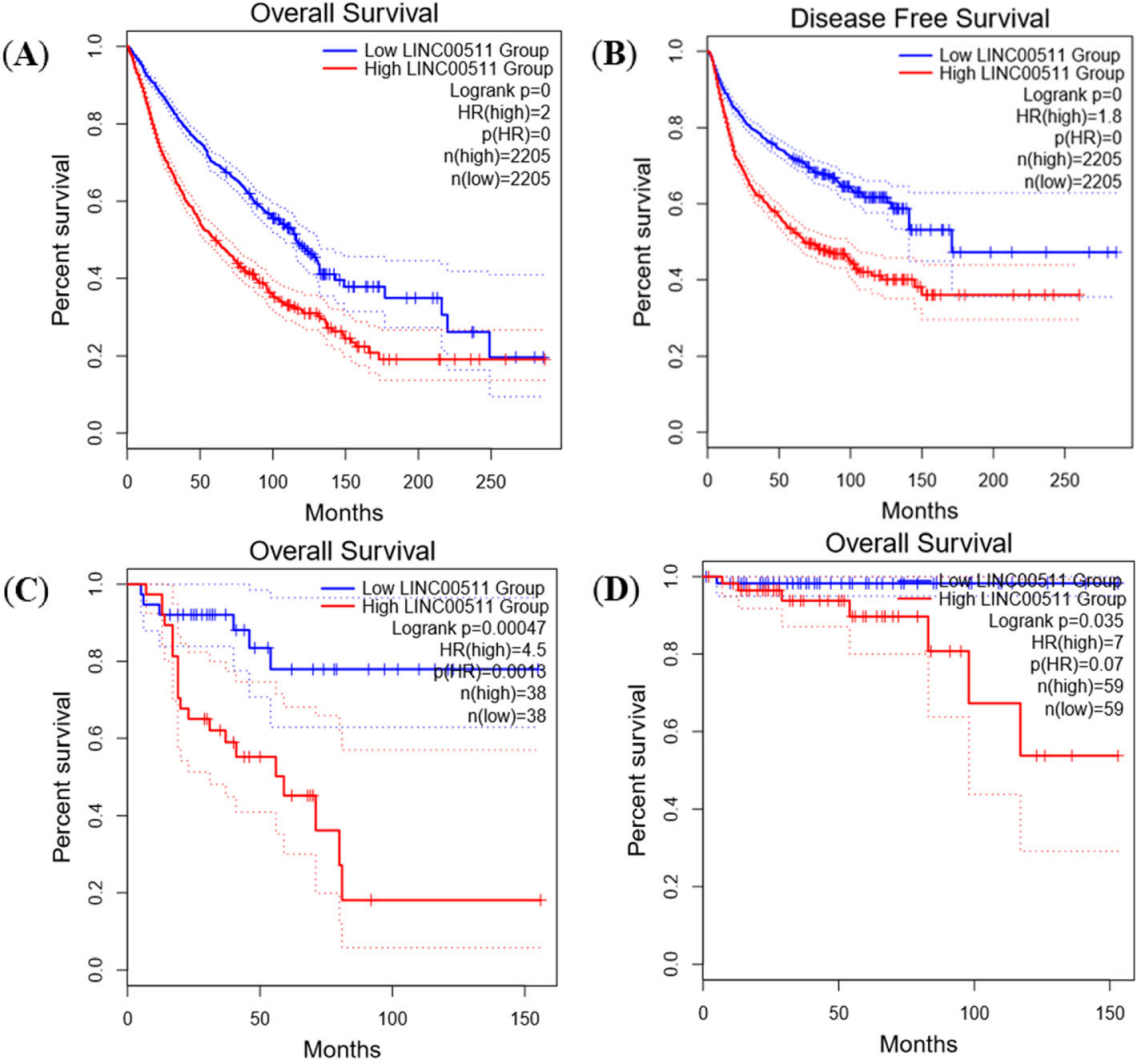
Kaplan–Meier plots depicting the prognostic potential of LINC00511 for cancer patients’ survival. **a** Overall survival plot. **b** Disease-free survival. **c** Overall survival plot for adrenocortical carcinoma (ACC). **d** Overall survival plot for thymoma (THYM)

To strengthen the results of our meta-analysis, we used GEPIA web server which contain a larger amount of sample size and performed bioinformatics analysis. The expression level of LINC00511 was analyzed in eleven different type of cancers (cancer types included in the meta-analysis). As shown on the Fig. 6, LINC00511 was highly expressed in almost all the related cancers, including lung adenocarcinoma (LUAD), lung squamous cell carcinoma (LUSC), pancreatic adenocarcinoma (PAAD), breast invasive carcinoma (BRCA), cervical squamous cell carcinoma (CESC), liver hepatocellular carcinoma (LIHC), kidney renal clear cell carcinoma (KIRC), kidney renal papillary cell carcinoma (KIRP), kidney chromophobe (KICH), glioblastoma multiforme (GBM), and brain lower grade glioma (LGG) (log_2_FC value > 1 and *p*-value < 0.01). The Fig. 7 shows the Kaplan-Meier curves of the overall survival (Fig. 7a) and disease-free survival (Fig. 7b) analyses. A total of 4410 patients were divided into LINC00511 low expression and high expression groups based on the median LINC00511 expression. The patients in high expression group showed a worst overall survival (HR = 2.00; log-rank *p*-value < 0.001) and disease-free survival (HR = 1.8; log-rank *p*-value < 0.001) than those in the low expression group. Furthermore, we explored the possibility for any association of the LINC00511 expression to overall cancer survival in other cancer types that are not involved in the meta-analysis. It was found that LINC00511 overexpression was significantly associated with worse OS in adrenocortical carcinoma (ACC) (HR = 4.5; log-rank *p*-value = 0.00047; Fig. 7c), and thymoma (THYM) (HR = 7.00; log-rank *p*-value = 0.035; Fig. 7d). These results confirmed that LINC00511 is overexpressed in different types of cancers and this is correlated with poor survival. Of note, the GEPIA web server was accessed on December 28, 2019.

## Discussion

LINC00511, a newly identified lncRNA, has been reported to be upregulated and to have an oncogenic function in diverse cancers including lung cancer, breast cancer, pancreatic cancer, cervical cancer, liver cancer, ovarian cancer and glioma. Its underlying mechanisms of action include promotion of proliferation, tumorigenesis, cell cycle progression, invasion, migration, metastasis and chemoresistance and inhibition of apoptosis [16–22, 24]. More importantly, upregulated LINC00511 has been associated with prognosis, suggesting that it can represent a biomarker for prognosis in cancer patients. However, there is still discrepancy regarding the relationship between LINC00511 expression and clinical outcomes. In the present study, a first-time, comprehensive meta-analysis on the prognostic value of LINC00511 in diverse human cancers is presented. Collectively, 14 eligible studies were systematically included.

The results obtained from this meta-analysis have shown that upregulated LINC00511 is strongly predictive of poor overall survival of cancer patients. Moreover, the combination of multivariate analysis of four relevant studies showed that LINC00511 may serve as an independent predictor of poor overall survival for cancer patients. The subgroup analysis revealed that elevated LINC00511 expression correlated with poor overall survival independently to the tumor type, sample size, cut-off value and the data extraction method. Nevertheless, some subgroup analysis results should be taken with caution, due to the small number of studies included. Subsequently, these findings were corroborated by the results of TCGA analysis. Additionally, it was found that patients with elevated LINC00511 expression were more prone to worse clinicopathological features including larger tumor size, advanced clinical tumor stage, lymph node metastasis, distant metastasis and disease recurrence. Besides, high LINC00511 expression was found to be associated with poor disease-free survival and progression-free survival.

Taken together, the above findings lead to the suggestion that LINC00511 could serve as a potential biomarker and functional regulator in human cancers. Molecular mechanisms investigations highlighted that LINC00511 exerts its oncogenic function mainly by modulating microRNAs functions [42–44]. For instance, via its competing endogenous RNA activity on hsa-miR-29b-3p, LINC00511 induced the expression of VEGFA leading functionally to pancreatic ductal adenocarcinoma progression [20]. Similarly, Lu et al. [16] found that LINC00511 targeted the miR-185-3p/E2F1/Nanog axis in order to promote breast cancer tumorigenesis and stemness. In a recent study, LINC00511 was also found to foster the process of gastric cancer by targeting miR-625-5p/NFIX axis [43]. These mechanistic studies revealed that LINC00511 could act at transcriptional and post-transcriptional level. Moreover, LINC00511 was found to interact with a variety of signaling pathways including JAK2/STAT3 [45], Wnt/β-catenin [46], and PTEN/AKT/FOXO1 [47], in the pathogenesis of cancers.

LINC00511 has been demonstrated to be a poor predictor for both cancer recurrence and progression. These analogous outcomes imply that there might be similar LINC0511-dependent mechanisms underlying these two events. In particular, LINC00511 has been shown to induce radio-resistance in breast cancer resulting in recurrence and progression by regulating STXBP4 expression via miR-185 [28]. Similarly, the silencing of LINC00511 in cervical cancer cells enhanced cancer drug paclitaxel’s sensitivity, suppressed cell viability, cell proliferation, migration and invasion, and promoted apoptosis, thereby preventing progression and recurrence [24]. To achieve those functions, LINC00511 modulated the expression of related proteins, namely Bcl-2, Bax, cleaved caspase-3, metalloproteinases 2 and 9, multidrug resistance protein 1 (MRP1) and P-glycoprotein. The resistance to paclitaxel caused by LINC00511 was also found in breast cancer and is mediated via regulating miR-29c/CDK6 axis [23]. Du et al. found, in glioblastoma multiforme (GBM), that high LINC00511 expression was correlated with recurrence, and ectopic LINC00511 enhanced GBM cells proliferation, EMT, migration and invasion by sponging miR-524-5p to indirectly regulate YB1/ZEB1 [39].

A number of limitations should be addressed when considering the findings of the present study. Firstly, all the studies included in the meta-analysis have been conducted in China, narrowing the representativeness of the results. Secondly, there is a rather limited number of studies available on major cancers, such as lung neoplasms and brain neoplasms. Therefore, the results on subgroup analysis based on cancer types should be taken with cautions. Thus, more clinical studies would be necessary to better assess the relationship between LINC00511 expression and cancer patients’ clinical outcomes.

## Conclusions

In summary, this study demonstrates that the overexpression of LINC00511 can strongly predict a poor survival in cancer patients and that it is associated to large tumor size, lymph node metastasis, advanced clinical stage, distant metastasis and disease recurrence. LINC00511 can thus serve as prognostic biomarker for cancer patients. Nevertheless, further large-scale and high-quality studies from different ethnic background are needed to confirm the present analysis.

## Data Availability

All data are included in this article.

## Abbreviations

ACC: Adrenocortical carcinoma
BC: Breast cancer
BRCA: Breast invasive carcinoma
CC: Cervical cancer
ccRCC: Clear cell renal cell carcinoma
CESC: Cervical squamous cell carcinoma
CP: Clinicopathological parameters
DFS: Disease-free survival
GBM: Glioblastoma multiforme
GL: Glioma
GTEx: Genotype-Tissue Expression
HCC: Hepatocellular carcinoma
KICH: Kidney chromophobe
KIRC: Kidney renal clear cell carcinoma
KIRP: Kidney renal papillary cell carcinoma
LGG: Brain lower grade glioma
LINC00511: Long intergenic non-coding RNA 00511
LIHC: Liver hepatocellular carcinoma
LUAD: Lung adenocarcinoma
LUSC: Lung squamous cell carcinoma
N: Normal
NOS: Newcastle-Ottawa Scale
NSCLC: Non-small-cell lung cancer
OC: Ovarian cancer
OS: Overall survival
PAAD: Pancreatic adenocarcinoma
PC: Pancreatic cancer
PDAC: Pancreatic ductal adenocarcinoma
PFS: Progression-free survival
qRT-PCR: Quantitative real time polymerase chain
RFS: Relapse-free survival
T: Tumor
TCGA: The Cancer Genome Atlas
THYM: Thymoma
TPM: Transcripts per million
